# [Expression of Concern] Leptin promotes breast cancer cell migration and invasion via IL-18 expression and secretion

**DOI:** 10.3892/ijo.2025.5816

**Published:** 2025-11-04

**Authors:** Kuangfa Li, Lan Wei, Yunxiu Huang, Yang Wu, Min Su, Xueli Pang, Nian Wang, Feihu Ji, Changli Zhong, Tingmei Chen

Int J Oncol 48: 2479-2487, 2016; DOI: 10.3892/ijo.2016.3483

Following the publication of the above paper, it was drawn to the Editor's attention by a concerned reader that the first two lanes of the Actin blot in Fig. 1D looked strikingly similar to the Actin panels in Fig. 2E for the MDA-MB-231 cell line, In addition, the Actin panel in Fig. 4A (showing a time series) looked very similar to the Actin panel in Fig. 4B (showing different treatments). Upon analyzing the data independently in the Editorial Office, it came to light that there was an overlapping pair of data panels for the immunohistochemical data shown in Fig. 6C, such that data which were intended to show the results from differently performed experiments appeared to have been derived from the same original source, and data featured in Fig. 6D had subsequently appeared in a paper published in the journal *Tumor Biology* that was written by different authors at different research institutes.

The authors were contacted by the Editorial Office to offer an explanation for these possible anomalies in the presentation of the data in this paper, although up to this time, no response from them has been forthcoming. Owing to the fact that the Editorial Office has been made aware of potential issues surrounding the scientific integrity of this paper, we are issuing an Expression of Concern to notify readers of this potential problem while the Editorial Office conitnues to investigate this matter further.

